# Teleoperation system for multiple robots with intuitive hand recognition interface

**DOI:** 10.1038/s41598-024-80898-x

**Published:** 2024-12-04

**Authors:** Lucas Alexandre Zick, Dieisson Martinelli, André Schneider de Oliveira, Vivian Cremer Kalempa

**Affiliations:** 1 Department of Information Systems, Universidade do Estado de Santa Catarina (UDESC), São Bento do Sul, 89283-081 Brazil; 2 Graduate Program in Electrical and Computer Engineering, Universidade Tecnolágica Federal do Paraná (UTFPR), Curitiba, 80230-901 Brazil

**Keywords:** Autonomous navigation, Human–robot, Multi-robots, ROS, Teleoperation, Mathematics and computing, Computer science, Information technology, Software

## Abstract

Robotic teleoperation is essential for hazardous environments where human safety is at risk. However, efficient and intuitive human–machine interaction for multi-robot systems remains challenging. This article aims to demonstrate a robotic teleoperation system, denominated AutoNav, centered around autonomous navigation and gesture commands interpreted through computer vision. The central focus is on recognizing the palm of the hand as a control interface to facilitate human–machine interaction in the context of multi-robots. The MediaPipe framework was integrated to implement gesture recognition from a USB camera. The system was developed using the Robot Operating System, employing a simulated environment that includes the Gazebo and RViz applications with multiple TurtleBot 3 robots. The main results show a reduction of approximately 50% in the execution time, coupled with an increase in free time during teleoperation, reaching up to 94% of the total execution time. Furthermore, there is a decrease in collisions. These results demonstrate the effectiveness and practicality of the robotic control algorithm, showcasing its promise in managing teleoperations across multi-robots. This study fills a knowledge gap by developing a hand gesture-based control interface for more efficient and safer multi-robot teleoperation. These findings enhance human–machine interaction in complex robotic operations. A video showing the system working is available at https://youtu.be/94S4nJ3IwUw.

## Introduction

Robots are mechanical devices designed to perform repetitive tasks, reducing the need for human intervention^1^. With the advancement of technology, an increasing number of robotic equipment has been developed to operate in various scenarios ranging from industrial to residential spaces^2^. For instance, industrial robots are utilized in production lines, and repurposed as autonomous home assistants. Moreover, the range of environments and specific demands for each application has expanded. A recent example is the exploration of Mars using various planetary rovers, such as Curiosity, Opportunity, and Perseverance^3^. These mobile robots can navigate within an environment to perform desired tasks^4^.

Although robots can replace humans in many tasks, there are cases where computational capabilities are not yet sufficient or feasible for full autonomy^2^. Semi-autonomous and teleoperated robots have become increasingly prevalent in situations where human intervention is not always possible or safe. Semi-autonomous robots involve some level of decision-making by the machine, but human involvement is necessary in some parts of the process to exert direct control^5,6^. In contrast, teleoperated robots require direct intervention and are remotely controlled by a human operator using Wi-Fi, Bluetooth, or more complex connections like the internet^1,7^.

Teleoperated robotic models can operate in complex, minimally known, and even high-risk environments because they do not rely on complete mapping of the surroundings, given that an operator commands the decisions^8,9^. Because commands can be triggered via Internet connections, robots can be operated from a well-prepared work environment, making them convenient for dangerous and inaccessible tasks^1,4^, such as underwater and intra-volcanic exploration or bomb disposal^10,11^.

Establishing an operating tool is necessary to implement teleoperated robots^7^. The currently used operating tools include joysticks or other physical devices that require the operator to carry them^1^. In addition, these devices are expensive and specific to a single type of robot, making them difficult to maintain and replace^12^. In recent applications, gesture recognition using cameras and video sensors has been employed to address the complexity of equipment. However some limitations still exist in the functionality of these tools. For instance, some of them need constant interaction, or can control only one model at a time, or even cannot recognize the orientation of the model^1^.

This work proposes a novel teleoperation method for multi-robot systems that utilizes gesture recognition, enabling angle control and the simultaneous operation of multiple robots. This approach leverages the MediaPipe framework for intuitive control over robot navigation and orientation. Additionally, the implemented autonomous navigation allows the operator to issue high-level commands, while the system autonomously plans and executes optimal paths, thereby enhancing multitasking in dynamic and hazardous environments. This method improves operational flexibility and safety in industrial automation, exploration, and emergency response scenarios. A noteworthy feature is its scalability, allowing the simultaneous control of multiple robots, which can enhance efficiency in large-scale operations.

The robot used for the development of this study was TurtleBot 3^13^, which was chosen for its navigation capabilities. The gesture recognition relies on the MediaPipe computer vision framework^14^. This framework extracts data about points on the human body from video inputs. Development and testing were conducted in a simulated environment using the Gazebo^15^ and RViz^16^ applications.

The remainder of this paper is structured as follows: the next section discusses the “Related work” section. In the section titled “The AutoNav” section, the development of the algorithm is presented in detail. “Experiments and validation” section introduces the experiments and validations conducted, along with the obtained results. Finally, “Conclusion” section presents concluding remarks and discusses potential future work.

## Related work

Teleoperation systems for mobile robots have evolved significantly, providing valuable insights into the field. Galarza et al. (2023) discuss a virtual reality teleoperation system that enhances robot manipulation; however, it requires specialized equipment and demands constant commands from the operator, which can be a limitation in accessibility and user fatigue^17^. Martinelli et al. (2020) focus on a human–robot interface for remote control, demonstrating effective motion recognition through deep learning techniques. Nonetheless, this approach operates a single robot at a time and requires constant operator involvement, which can impact efficiency in scenarios involving multiple robots^1^. Shamshiri et al. (2024) introduce a teleoperation method that utilizes a digital shadow for path creation, which ensures a structured approach to robotic control but necessitates prior route planning, limiting adaptability in dynamic environments^18^.

Chen et al. (2024) propose GestureMoRo, an algorithm for autonomous mobile robot teleoperation based on gesture recognition. This system, while limited to controlling a single robot and requiring constant operator input, stands out for being tested on a real-world device^19^. Similarly, Pantusin et al. (2024) introduce a virtual teleoperation system focused on mobile manipulator robots for object transport and manipulation, which also necessitates specific equipment in the form of a haptic device^20^. Zaman and Wu (2023) explore hand gesture-based control of a Mecanum-wheeled mobile robot, but like other approaches, it controls only one robot at a time and demands a high level of operator interaction, which can reduce efficiency in prolonged operations^21^. These systems highlight the trade-offs between specialized hardware and operator involvement, emphasizing that while they achieve high precision and control, they may hinder scalability and accessibility in more generalized environments.

The proposed apprach aims to address these limitations by introducing a teleoperation method that utilizes gesture recognition for more intuitive control. By minimizing reliance on specific equipment and enabling the simultaneous operation of multiple robots, this approach seeks to enhance efficiency and adaptability in various operational contexts.

## The AutoNav

**Fig. 1:**
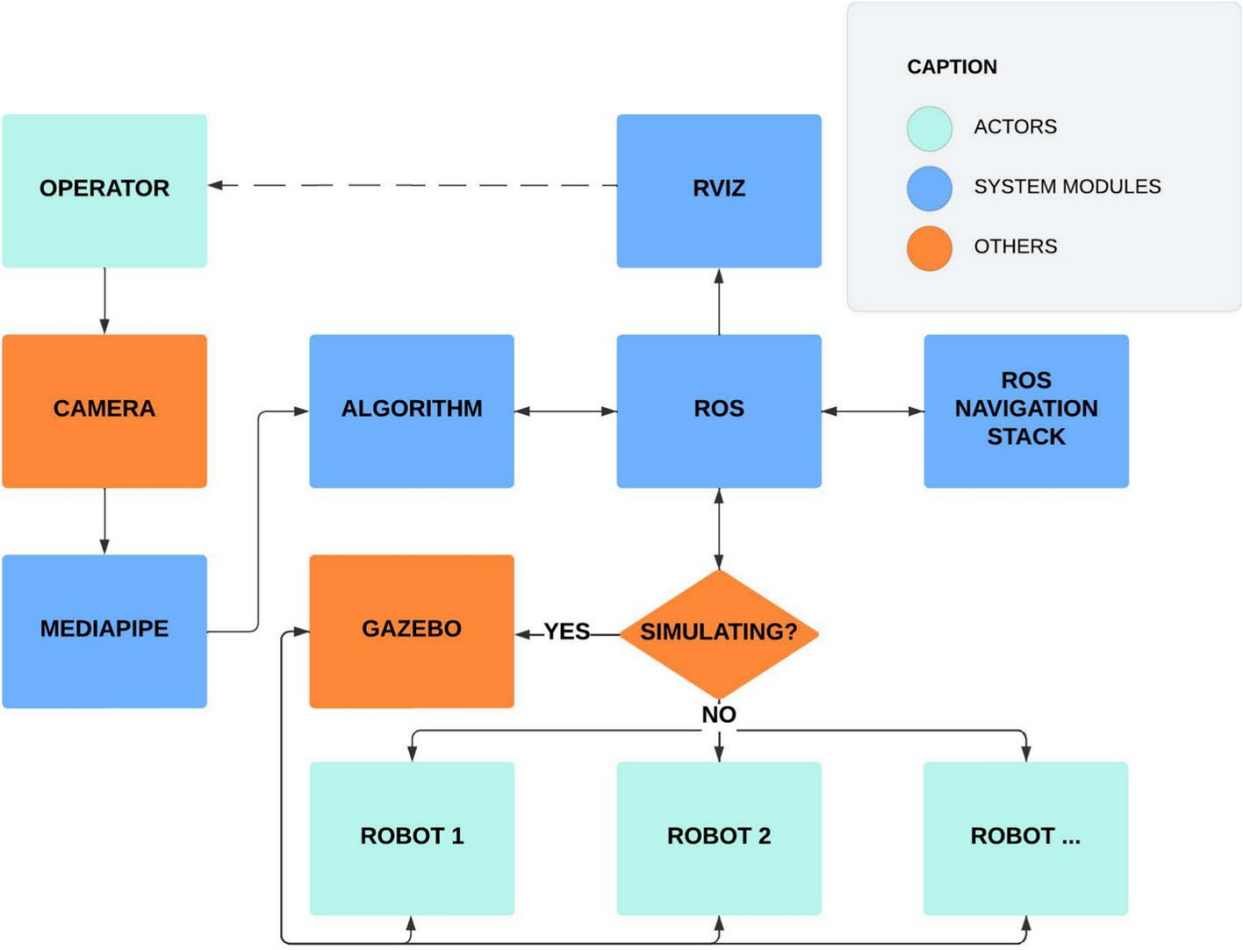
Overall of AutoNav approach.

This section presents the proposed AutoNav, a multi-robot teleoperation algorithm based on autonomous navigation and gesture commands captured through a USB camera. The robots interact through wireless connections directed by the Robot Operating System (ROS) through topics. The interface was implemented in the RViz application, a 3D tool for displaying robot models, sensor data, and spatial transformations in three-dimensional environments^16^. Two independent markers signal the position and state of the operator’s hand and the desired positions of the robots. Data regarding the operator’s hand position were extracted using MediaPipe, which received video input from a USB camera connected to a computer. Based on this input, users can send new destinations to robots, specifying the final position and angle, with autonomous navigation executed by ROS Navigation Stack^22^. Figure 1 summarizes components and data flow in the proposed strategy.

**Fig. 2:**
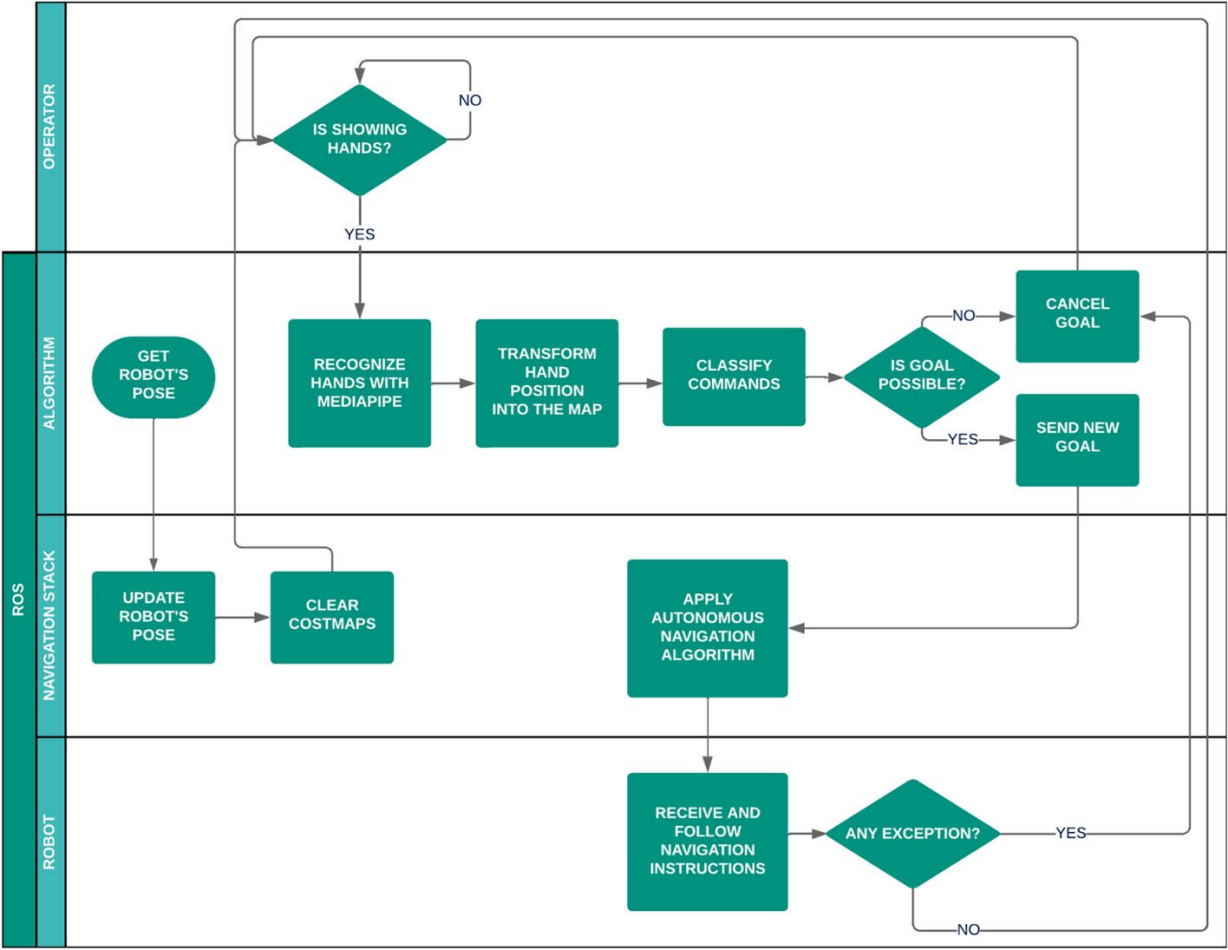
Agents, processes, and key decision points for proposed approach.

Figure 2 illustrates the interactions of the system, with each agent represented by specific lanes. Information flows unidirectionally between components, showing a clear sequence of operations. This cyclical model allows continuous information updates. The algorithm receives data from the operator’s hands via MediaPipe, fed by the camera. The algorithm then communicates with the robots and RViz using ROS, enabling the operator to visualize the execution time information provided by the system and initiate a new cycle after each interaction.

The development of AutoNav prioritized ethical considerations, with a focus on ensuring the safety of human operators and bystanders throughout the teleoperation process.

### Recognition and capture of hand points

Hand gesture recognition is fundamental in creating more intuitive and natural human–machine interactions^23^. Integrating gesture recognition methods with other techniques significantly enhances user experience, making it more dynamic and seamless. In this context, this research aims to operate the robot based on hand gesture recognition, eliminating the need for specific equipment and relying solely on a USB camera as the data input source.

The performance of hand pose recognition plays a crucial role in the overall functionality of the system. Accurate detection ensures that the robot can respond to user commands with precision^1^. However, inaccuracies or delays in recognizing hand gestures can lead to misinterpretations, causing unintended robot movements. Therefore, the system’s stability and reliability heavily depend on a robust and consistent recognition framework that minimizes errors and maintains smooth interaction throughout the process.

**Fig. 3:**
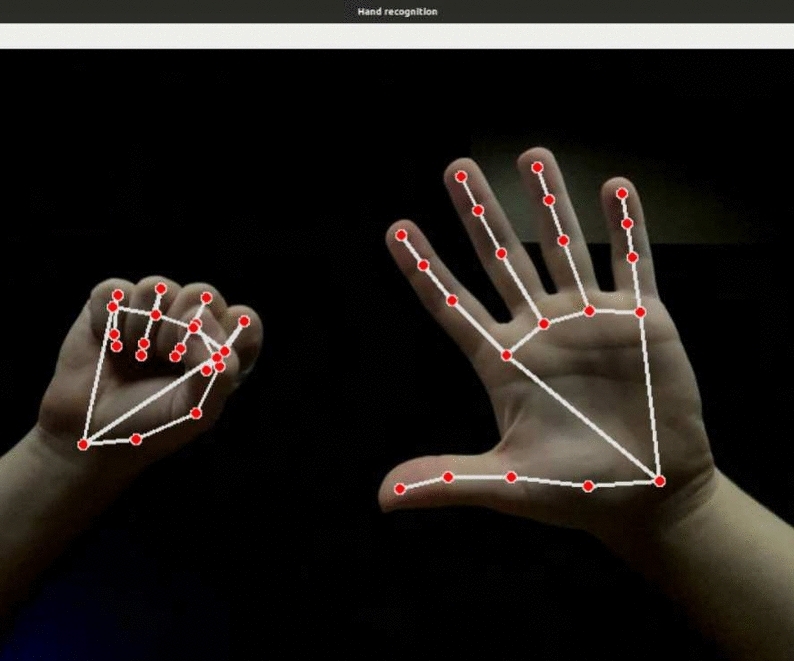
Hand points recognition with MediaPipe.

MediaPipe is an open-source library developed by Google and designed to perform precise and efficient hand tracking^14^, as illustrated in Fig. 3. Hand-position reading using MediaPipe is based on deep learning and computer vision techniques. The library implements a trained neural network to detect hands in an image or video frame. If detected, MediaPipe defines 21 key points for each hand, referred to as landmarks, representing various parts of the hand anatomy, such as the fingertips, joints, and palms^1,24^. These landmarks are then tracked during execution, allowing the system to accurately follow the position and orientation of the moving hand relative to the camera.

**Fig. 4:**
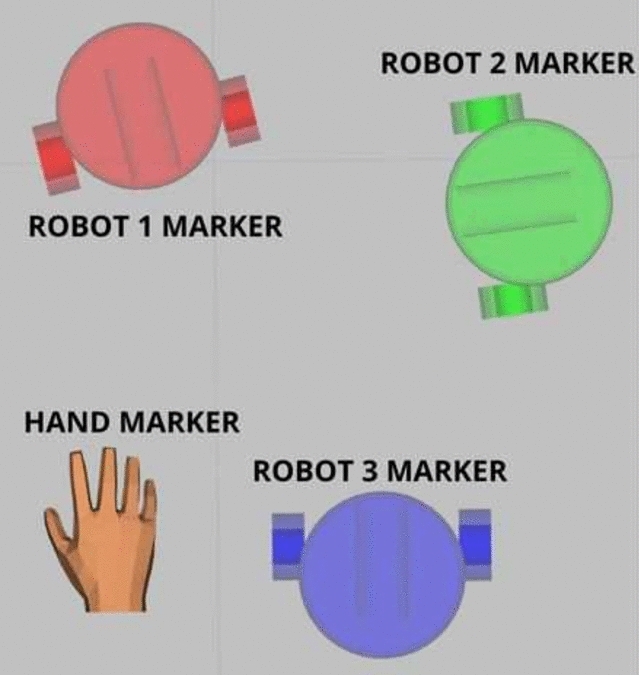
Markers representation.

In the proposed method, the state of the hand, whether open or closed, is determined by evaluating the average distance of the operator’s five fingers. The ‘closed_hand_threshold’ variable in the system helps gauge the user’s distance from the camera to distinguish between open and closed hand gestures. When the average distance exceeds the threshold, the hand is classified as open; otherwise, it is classified as closed. This approach allows the operator to capture moments of hand state changes and provides visual feedback at the interface.

A grid method was employed to accurately determine the hand’s location relative to the map by dividing the camera-captured image into a mesh of points. This method allows images of different sizes to be translated to any map size^25^. The algorithm identifies the hand’s position in the camera image, maps it to the corresponding coordinates on the map, and transmits this data for runtime visualization in the RViz interface.

Markers guide the user visually and work as follows. The hand-shaped marker serves as a cursor. It is positioned on a map, proportional to the operator’s hand position in the camera video. Although the markers for the robot positions are initially set in the algorithm by receiving the current positions of the robots, they can be moved to other points by the user using the hand states. An amplified representation of these markers is shown in Fig. 4.

When teleoperated, the markers for robot positions function as goal indicators. Once ‘released’ (when the hand carrying a marker returns to the ‘open hand’ state), the algorithm takes the marker point as the desired position and begins autonomous navigation until the desired position is reached or a new position is specified.

The following technique enhanced the accuracy and consistency of picking up and releasing the markers. When the operator’s hand is open, the action is exclusively directed toward navigation, and the robot marker remains static. Similarly, when the hand is in a closed state, the priority is to maintain the hand as a cursor. Therefore, to simulate the action of picking an object, the robot marker starts following the hand only when it transitions from the open to the closed state. Similarly, the marker is released when the hand transitions from closed to open. This approach avoids inconsistencies in operation, such as the closed hand overlaying multiple markers and triggering the pickup action improperly or interrupting the action owing to instability in reading hand points. This strategy provides more consistent and accurate operation, ensuring process integrity.

### Virtual experimentation

**Fig. 5:**
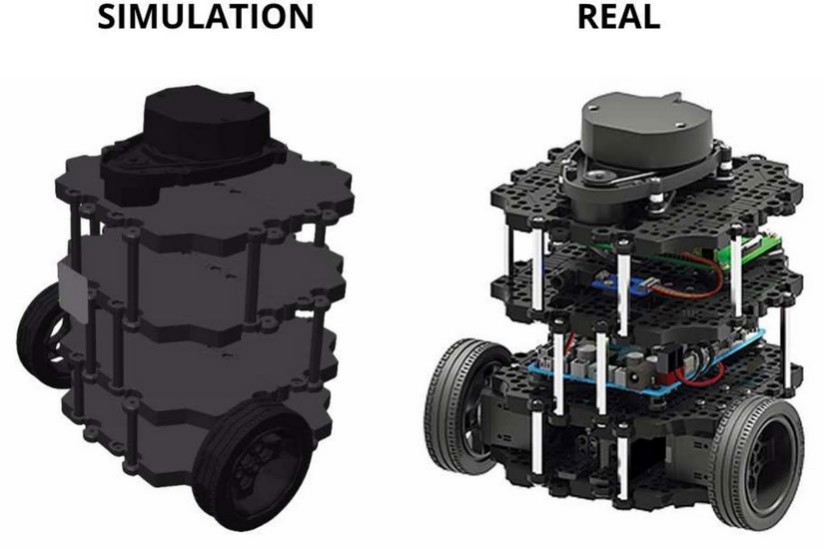
TurtleBot 3 Burger model in both simulated and real environments^13^.

The TurtleBot 3 Burger was selected for this project due to its reputation as a classic and accessible mobile robot. Its affordability and compatibility with current simulators, such as Gazebo and RViz, make it a practical choice for the development^26^. The TurtleBot 3, manufactured by Robotis, features a cylindrical shape, weighs around 1 kg, and is equipped with components such as the LDS-01 laser distance sensor, a 3-axis IMU, and a Raspberry Pi as its main SBC^13^. Figure 5 shows the model in both simulated and real environments.

Gazebo and RViz were chosen as the simulation tools for their strong integration with ROS and their open-source availability. Gazebo simplifies TurtleBot 3 integration^26^, while RViz efficiently handles sensor data visualization. Launchers were created to ensure consistent and flexible initialization of both the environment and algorithm. A detailed practical explanation of the virtual experimentation is available on AutoNav’s GitHub repository (address in code availability statement section). The setup allowed efficient simulation of maps and testing through the ROS topic-based communication system, ensuring seamless data transmission between the components^27^.

### Navigation strategy for multi-robots

Navigation strategy plays an essential role in the scope of this study, efficiently replacing human decisions to achieve autonomous navigation of multi-robots^28^. This development employs the ROS Navigation Stack, a modular tool integrated into the ROS as the primary solution for attaining autonomous navigation of a fleet of robots.

**Fig. 6:**
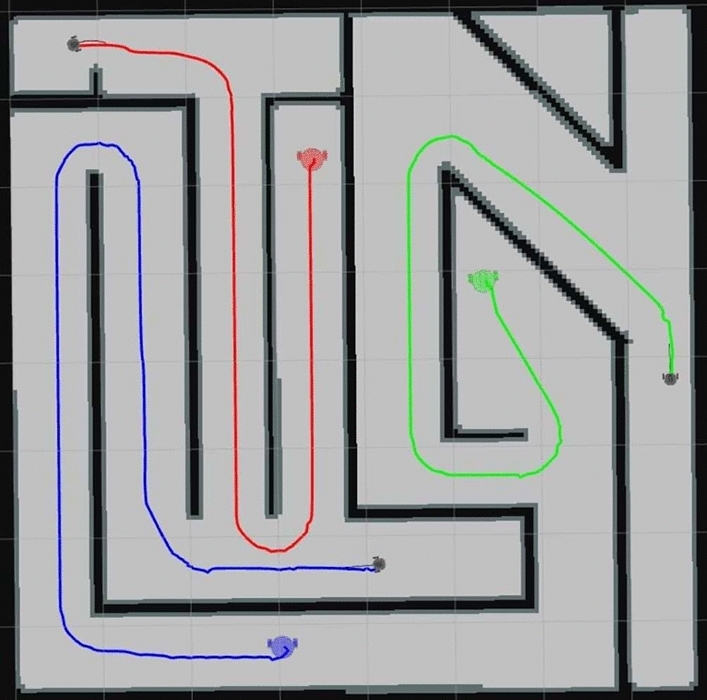
Interface with autonomously generated navigation routes by the algorithm.

Effective route planning within the ROS Navigation Stack involves intricate interactions between local and global planners^29,30^. Each local planner uses techniques such as the dynamic window approach algorithm to make short-term decisions based on the dynamically changing local environment and capabilities of each robot^31^. Concurrently, the global planner employs algorithms such as A*^32^ or Dijkstra^33^ to focus on long-term strategies and planning trajectories to connect starting points to final destinations for multi-robots, disregarding future environmental changes^34,35^. This hybrid approach, blending short- and long-term strategies, enables the ROS Navigation Stack to generate efficient routes, adapt to unforeseen circumstances, and maximize the effectiveness of autonomous navigation for a fleet of robots. Examples of generated routes are shown in Fig. 6.

The cost map, responsible for creating a representation of the collective environment surrounding the fleet of robots, distinguishes accessible areas from obstacles^27^. The dynamic cost map adapts to the evolving surroundings and is employed as a shared memory for all robots, facilitating collaborative route planning^36^.

### Integrating hand gestures and multi-robots teleoperation

Initially, computer vision using MediaPipe takes center stage in the application by identifying and classifying hand key points. Detecting these key points forms the foundation of teleoperation, as gesture recognition makes the interface between the user and robotic models fluid and intuitive^25^. The algorithm collects, analyzes, and transforms these points into coordinates to represent the markers in RViz.

**Fig. 7:**
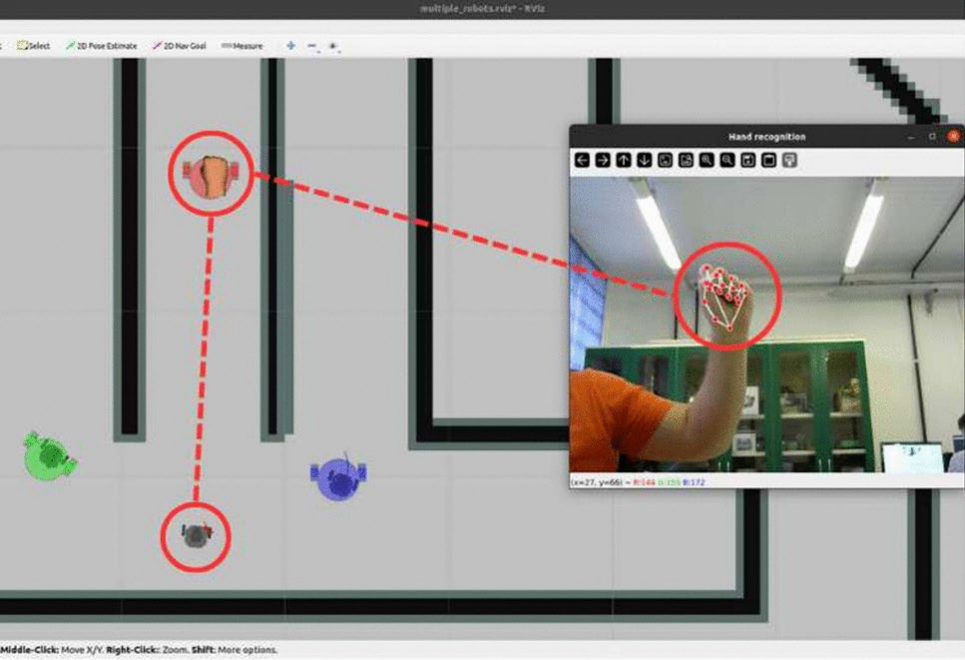
System interface with visualization of hand points capture on the operator’s camera.

Markers are created on the RViz interface to generate a dynamic graphical representation of multi-robot cursors. These markers display the hand’s reading position and state as well as the current and desired positions of each robot. In addition, they dictate situations where interaction between the hand and robots can occur. The objective model derived from a marker provides the operator free time while allowing destinations to be redefined at any moment for each robot. Furthermore, they enable the integrated visualization of robots and their interactions. Although each robot is tracked by Gazebo and the hand by a MediaPipe video, RViz serves as the system’s primary interface with unified information. The interface of the operator used for multi-robots is shown in Fig. 7.

Considering the complexity and cost, multiple TurtleBot 3 robots were developed and tested in a simulated environment on the Gazebo platform. This platform provides robots with physical conditions similar to a real environment and allows for the visualization of detailed models.

## Experiments and evaluation

This section describes the experiments and validation conducted in this study. The validations focused on testing the operator’s operation time and verifying user preferences. In addition, data on the number of collisions during the trajectories were collected.

The developed system was compared with a conventional teleoperation model, where the operator controls the robot with a joystick, to provide a comprehensive and unbiased analysis of different robotic teleoperation contexts. The proposed and conventional models were named AutoNav and ManualNav, respectively. Various participants representing a range of profiles were included in the tests without specific selection. The operators received brief instructions on the methods used.

Two limitations should be acknowledged. Firstly, the study did not assess the operators’ prior knowledge in the studied contexts. Secondly, only a single map was utilized, potentially limiting the generalizability of findings to different environments.

### Operation experiments

The first test evaluated differences in the operation time required for each tested method. For each operation, the algorithm recorded the total execution and the operation time, which comprised the entire duration during which the user interacted with the system. From these data, the free time for each operation was calculated, which, in real-world scenarios, can be utilized productively for other tasks. A deliberate sample of 11 operators was selected as the study population to ensure validity. Although the choice was not random, specific criteria regarding age and sex were not predefined during the selection process, ensuring a diverse sample.

Two sets of tests were conducted for each operator, one for each teleoperation method, with three robots and three objectives. This allowed the investigation of the particularities of each method and the assessment of the impact of environmental differences, considering that the routes had unique characteristics.

**Table 1:** Results regarding the operation time tests.

Operator	Age	Gender	AutoNav				ManualNav			
			Total time	Op.time	Free time	Free time (%)	Total time	Op.time	Free time	Free time (%)
1	19	M	221.27	20.94	200.33	91	464.56	435.48	29.08	6
2	21	M	250.2	14.79	235.41	94	412.06	386.72	25.34	6
3	19	M	218.39	16.69	201.7	92	591.38	518.73	72.65	12
4	22	M	214.88	23.32	191.56	89	402.53	391.24	11.29	3
5	31	M	233.72	26.42	207.3	89	387.44	371.89	15.55	4
6	21	M	207.16	30.14	177.02	85	403.19	388.37	14.82	4
7	28	F	209.72	24.09	185.63	89	404.71	384.66	20.05	5
8	26	M	222.08	20.17	201.91	91	501.98	479.91	22.07	4
9	22	M	227.79	29	198.79	87	355.03	342.41	12.62	4
10	29	M	219.31	23.98	195.33	89	441.42	428.87	12.55	3
11	54	F	241.54	40.92	200.62	83	414.69	403.72	10.97	3
		Average	224	25	200	89	434	412	22	5

The total execution time was extracted from the difference between the timestamp at which the operator signals the completion of the execution and at which the algorithm starts, thus encompassing a small time margin before and after execution^1^. The operation time was obtained by summing the set of intervals during which the operator interacted with the system. The free time for each execution is calculated by subtracting the operation time from the total execution time. Measured values for all operations are listed in Table 1.

**Fig. 8:**
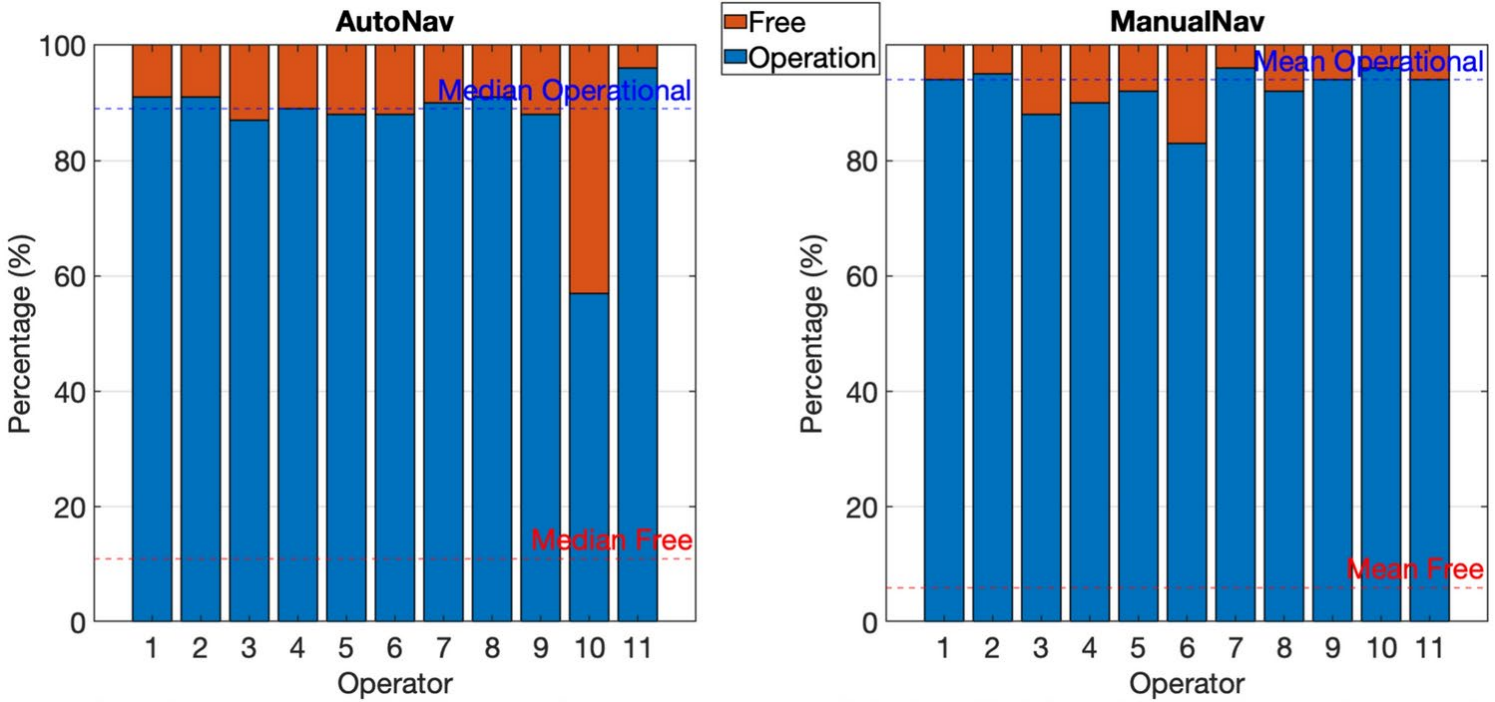
Graphical analysis of operation experiments.

Based on the tests, a noticeable difference in the operators’ free time was observed. While operations in ManualNav practically consumed the entire execution time, operations with AutoNav remained stable at around 23 seconds, reaching up to 94% of free time for experienced users. It is important to consider that free time is calculated based on the user’s interaction with the command model, implying the possibility of a greater difference in real demands due to the constant command provided when using ManualNav, as illustrated in Fig. 8.

Moreover, the findings reveal approximately 50% reduction in the total execution time of AutoNav compared to ManualNav. This improved outcome is expected because the operator loads the robot markers to the desired destinations during a pre-navigation time interval, eliminating the need for decision-making during operation. Additionally, efficiency gains stem from the simultaneous execution of multiple operations, which cannot be achieved through conventional remote-control methods. Moderate driving maneuvers performed by the robots contributed to this time reduction because they followed a predefined path and maintained a safe distance from the walls during turns.

### Collision experiments

**Fig. 9:**
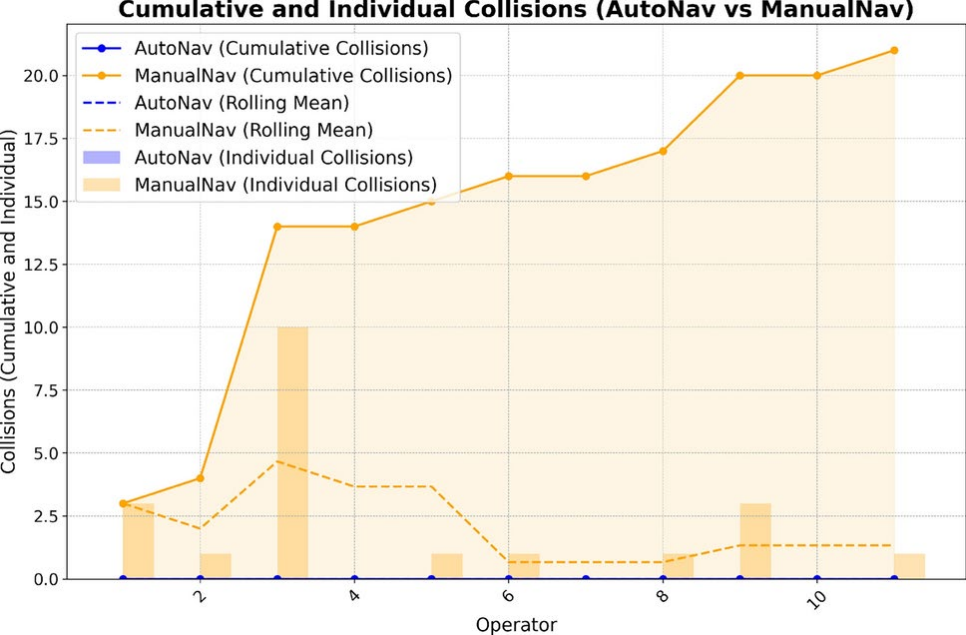
Results regarding the quantity of collisions during operations.

For the collision quantity test, the system counted the number of collisions during execution. The collision indication is obtained from RViz, which logs the sensor data and provides the distance to the nearest wall. This test was run concurrently with the operation time test for realistic and practical data. A graph depicting the number of collisions during operation is shown in Fig. 9.

A noticeable difference in the number of collisions is observed when using the two methods. With ManualNav, 21 collisions were recorded in 11 operations. In contrast, no collisions occurred when using AutoNav. This outcome was predictable because of the collision-prevention methods provided by the ROS Navigation Stack library.

### Usage preference

This study verified user preference through a questionnaire after the completion of operations. Each operator completed only the data necessary for the experiments and indicated which method best met their expectations in terms of four categories: comfort, practicality, precision, and efficiency.

**Fig. 10:**
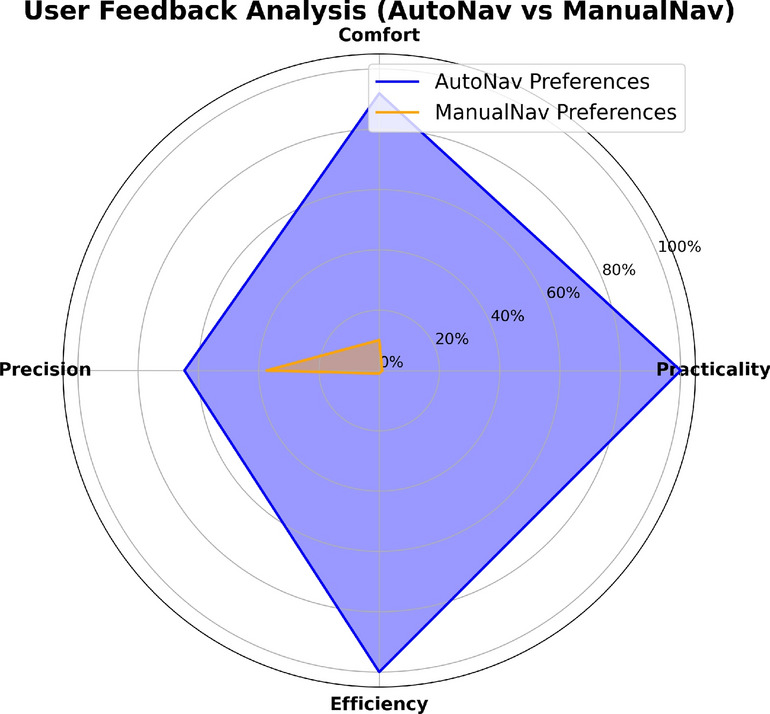
Results of the survey on operators’ usage preference.

In addition to direct and generalized responses, operators were allowed to include an analysis of the main differences perceived during usage. This approach focuses on the intrinsic details that are not perceptible through visual analysis, allowing the operator to describe their perception after use. The survey results are shown in Fig. 10.

The results reveal that AutoNav was satisfactory in all aspects compared to ManualNav, as illustrated in the radar chart. In this chart, each axis represents a specific criterion, and the closer a data point is to the outer edge, the better the performance in that category. Notably, 100% of the operators prefer the proposed algorithm in terms of practicality and efficiency. Moreover, 90% of participants opted for AutoNav when addressing comfort during handling. Regarding precision, the number drops to approximately 63% because, according to evaluations from some operators, the ability to define the path on its own makes the conventional method more precise.

Among the submitted textual evaluations, considerations related to both methods are listed, with the advantage of the proposed algorithm over the conventional one mentioned repeatedly. The operators appreciated the capability of the proposed algorithm to allow them to detach from the equipment and perform other tasks after being sent to the desired destination. However, a major disadvantage is the lack of constant control, indicating the need, in some cases, to relocate the marker to make slight adjustments to the sent path. Furthermore, an operator affected by repetitive strain injuries (RSI) preferred the proposed algorithm, stating difficulties associated with prolonged manual management due to the RSI condition.

For further insights, the evaluations can be accessed in both English and their original language in the data repository used for this study.

### Overall evaluation

The evaluation of the proposed approach compares the developed system, AutoNav, with a conventional teleoperation model referred to as ManualNav, in which the operator controls the robot using a joystick. This comparison aims to provide a comprehensive and unbiased analysis across various robotic teleoperation contexts, considering the intuitive aspects of multi-robot teleoperation.

The analysis is conducted from several perspectives. The operational experiments evaluate the differences in task execution time and available free time. In collision experiments, we analyze the performance of untrained operators regarding the number of collisions during task execution. Finally, we examine usage preferences by having operators share their experiences with both approaches.

These comprehensive analyses allow us to compare the proposed AutoNav approach with the standard Manual approach, as illustrated in Table 2. The AutoNav system shows significant improvements across all metrics compared to the standard manual method.

**Table 2:** Overall comparison of AutoNav and ManualNav.

Analysis	AutoNav	ManualNav
Operation (Free time)	89.00%	5.00%
Collision (Mean/Operator)	0	1.61
Usage (User preference)	88.65%	11.37%

## Conclusion

This work contributes to the research on mobile robotics with the application of autonomous navigation, focusing on the human–robot interface used for multi-robot teleoperation. An architecture that integrates autonomous navigation with point capture was selected for practical and affordable teleoperation.

Currently, acquiring specific equipment for robotic control is optional. Similar results can be achieved at a lower cost using a USB camera for image recognition.

Tests and validations were conducted to prove the effectiveness of the proposed algorithm. The proposed model reduces the total execution time by 50% and increases the operator’s free time during execution compared to conventional teleoperation models. This increase can reach more than 94% of the total time for long routes, providing convenience and comfort to the operator. Moreover, collision and time tests demonstrated the efficiency of applying autonomous navigation in the algorithm, with a 100% reduction in collisions compared to the conventional model.

The final validation was conducted using the operators in the previous procedures, where each received a form indicating their preferences for each of the four mentioned aspects. In addition, operators could provide a brief analysis of their experiences with each model along with the forms. The research results reveal a 100% preference among operators for the proposed algorithm in terms of efficiency and practicality and a 90% and 63% preference for comfort and precision, respectively.

The analyses identified reduced effort required by the proposed algorithm to perform tasks and precision limitations owing to gesture-based control. Furthermore, the application was efficient for operators with RSI because of its quick and efficient method of selecting the destination.

Consequently, the benefits of concurrently implementing computer vision and autonomous navigation for a more intuitive operator experience are evident. It ensures the safe completion of routes and offers objective advantages in the logistics process in outdoor environments, along with considerable cost reduction, avoiding unnecessary acquisition of specific equipment for robot operation. Additionally, there are safety benefits for the operator, who can perform operations in a prepared environment, free from external risks. Moreover, reallocating the operator time allows them to operate multi-robots simultaneously or perform other tasks while the robot completes its trajectory. Finally, reducing the collision rate per operation is beneficial in terms of the frequency of maintenance and replacement of the robotic equipment.

### Future work

In the future, it will be noteworthy to transition this algorithm from a simulated to a real-world scenario by implementing it in physical robots. Practical validation can provide unique insights into the effectiveness and adaptability of the algorithm under real-world conditions. Moreover, an additional innovation would involve transforming the control vector into a manipulable object, allowing operators to control the physical objects carried by the robots to set new destinations. This approach decouples the robot teleoperation action and focuses on object control, relevant to the practical demands of mobile robotics in real-world situations.

## Data Availability

The datasets generated and analyzed during the current study are available in the GitHub repository, https://github.com/LucasZick/auto_nav/blob/Main/results.

## Code availability

The code for the main application developed and discussed in this study is available in the GitHub repository, https://github.com/LucasZick/auto_nav/tree/Main.
